# Catch-Up Growth as a Risk Factor for Rapid Weight Gain, Earlier Menarche and Earlier Pubertal Growth Spurt in Girls Born Small for Gestational Age (SGA)—A Longitudinal Study

**DOI:** 10.3390/ijerph192416808

**Published:** 2022-12-14

**Authors:** Magdalena Durda-Masny, Weronika Stróżewska, Anita Szwed

**Affiliations:** Institute of Human Biology and Evolution, Faculty of Biology, Adam Mickiewicz University, Uniwersytetu Poznanskiego 6, 61-614 Poznan, Poland

**Keywords:** small for gestational age, SGA, AGA, catch-up growth, body mass index, puberty, menarche, adolescent growth spurt

## Abstract

Most children born small for gestational age (SGA) have rapid postnatal growth. Despite its positive aspects, catch-up growth may affect the level of adipose tissue in the pre-pubertal and pubertal periods and therefore affect the age of puberty. The aim of this study was to determine the associations between size at birth, catch-up growth in infancy, BMI in peripubertal period, age at menarche, and the parameters of adolescent growth spurt of body height in girls born SGA. For 297 girls (22.6% SGA; 77.4% appropriate for gestational age (AGA)) complete body weight and height measurements and age at menarche were obtained. Adolescent growth spurt parameters were estimated using the JPA2 model (AUXAL SSI 3.1). Calculations were made in the Statistica 13 program using the Kruskal-Wallis and Kaplan–Meier tests. Girls born SGA with catch-up had the highest BMIs at the age of 8 years (H = 94.22, *p* < 0.001) and at menarche (H = 58.21, *p* < 0.001), experienced menarche earliest (H = 21.77, *p* < 0.001), same as the onset (H = 6.54, *p* = 0.012) and peak height velocity (H = 11.71, *p* = 0.003) of their adolescent growth spurt compared to SGA girls without catch-up and AGA girls. In SGA girls, catch-up growth has far-reaching consequences such as increased risk of fat accumulation and a rapid transition to puberty.

## 1. Introduction

Small for gestational age (SGA) is defined as a birth weight or length below the 10th or 3rd percentile, or below −2 standard deviations (SD) from the mean for weight and/or length for the newborn’s gestational age [1,2]. Approximately 3–11% of live-born newborns are born SGA [3]. As compensation for prenatal growth retardation, SGA children may be more likely to have rapid postnatal growth known as catch-up growth and can achieve a height and body mass >−2 SD [4]. Catch-up growth occurs in early postnatal life and, in most children, is completed by the age of 2 years [5,6]. The term catch-up growth has been introduced by Prader and colleagues [7] to describe the phase of rapid growth following a transient period of growth inhibition that allows a child to accelerate toward his/her original pre-retardation growth curve [8,9]. On one hand, catch-up growth has advantages in terms of body growth, immune response, and neurodevelopment [10]. On the other hand, low birth weight resulting from intrauterine growth restriction and the presence of catch-up growth in infancy result in an increased risk of obesity and becoming overweight in children [11]. Therefore, children born SGA are particularly at risk of becoming overweight or obese. A similar correlation has also been shown in children born prematurely [12]. It has been documented that childhood obesity is associated with the increased incidence of metabolic disorders later in life [13]. In addition to this already well-known relationship, the increased level of adipose tissue in children may affect the rate of puberty and distort the course of linear growth in adolescence [14]. The adipose tissue exhibits endocrine activity, which may interact with sex hormones or insulin-like growth factor 1 (IGF-1) [15,16]. Hence, being born as SGA is associated with increased long-term risks for developing insulin resistance, type 2 diabetes [17], metabolic syndrome [9,18], hypertension [19], and cardiovascular diseases [20], which fits in the *thrifty phenotype hypothesis* proposed by Barker [21,22]. At the same time, lack of catch-up growth and persistent poor postnatal growth are associated with short stature, more frequent infections, and impaired cognitive development [23]. Approximately 10% to 15% of children born SGA have persistent growth failure beyond two to three years of age [24,25]. The mechanism determining the appearance or absence of catch-up growth in SGA children is still uncertain [3]. However, a decrease in insulin sensitivity and disturbances in the growth hormone (GH)—IGF-1 axis are considered important results of fetal programming [10].

In general, the relationship between birth weight and the course of physical development of children in the later stages of life has been confirmed in many previous works [26,27,28,29]. In addition, many studies have proved the increased risk of obesity and metabolic syndrome later in life for children born SGA. However, studies on the relationship between SGA and its far-reaching consequences for the linear growth and rate of puberty are sparse, and the results of these studies are inconsistent [30,31,32,33,34,35]. Moreover, most studies did not distinguish between those SGA children who developed catch-up growth and those who did not [4,23,31,36,37,38]. Due to the lack of studies that would comprehensively analyze the relationship between the occurrence or lack of catch-up growth and a set of parameters defining BMI, puberty, and linear growth rates in adolescence in girls born SGA, our goal was to analyze the relationship between catch-up growth, rate of weight gain in peripubertal period, timing of puberty and the parameters of the pubertal growth spurt of the body height in SGA girls.

## 2. Materials & Methods

### 2.1. Participants and Study Design

**Figure 1:**
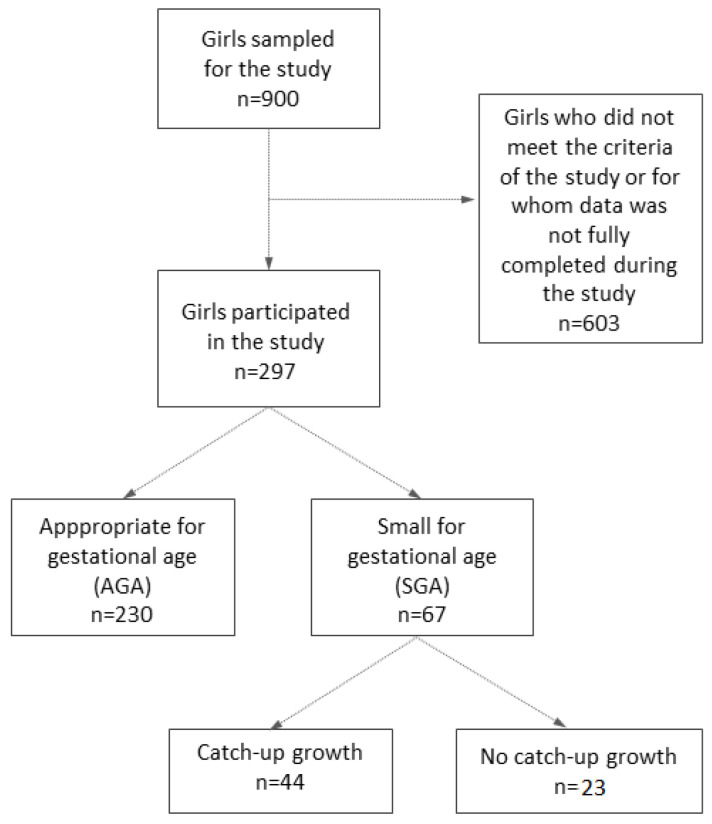
Flow chart of the study group and its division according to the size at birth and the occurrence of catch-up growth.

The ongoing study was established in 2008 and enrolled 900 girls attending randomly selected primary schools in the Wielkopolskie voivodeship, which is located in the central-western part of Poland. All girls in the cohort were Caucasian. The girls were 10 years of age at the start of the study, and measurements of body mass and height were repeatedly performed until the age of 16. The main inclusion criteria for the studies were a birth weight > 1500 g and <4000 g and a gestational age of 37 + 0 to 42 + 0 weeks. We excluded girls diagnosed with diseases that may disturb the process of growth and puberty. The study did not qualify, among others, children affected by chromosomal defects, endocrine disorders related to the abnormal secretion of growth hormones or sex steroids, or diseases that require long-term treatment with glucocorticosteroids. Informed consent was obtained from all the subjects involved in the study as well as from their parents or legal guardians. The final sample consisted of 297 girls (Figure 1). The number and percentage of girls in categories of socioeconomic status are presented in Table 1. This research has been approved by the Bioethics Committee at Poznan University of Medical Sciences (reference number: 29/10).

### 2.2. Size at Birth

Data on birth weight and gestational age were obtained from child health record books. The Fenton infant preterm growth chart for girls [39] was used to assess the relation of birth weight to gestational age, and thus assess size at birth. Girls were categorized as SGA when their birth weight was below the 10th percentile for their gestational age, whereas girls born with a body mass above the 10th percentile for their gestational age were categorized as appropriate for gestational age (AGA). In our study, AGA girls were a control group. Girls born as large for gestational age (LGA), i.e., those born with birth weight > 4000 g [40], were considered nonparticipants.

### 2.3. Anthropometric Measurements

To assess the occurrence or absence of catch-up growth and the parameters of adolescent growth spurt as well as body mass index in the later stages of the girls’ life, several measurements of body height and weight were performed. From the beginning of the study—that is, from the age of 10—the girls’ heights and weights were measured at annual intervals. Measurements were performed in the school nurses’ offices by medical personnel trained by anthropologists. Body height was measured with a GPM anthropometer with a measurement accuracy of 1 mm with the subject standing barefoot with the head adjusted such that the Frankfurt plane was horizontal [41]. Weight was measured on a medical scale with an accuracy of 100 g. Data on the weight and height from birth to two years of age were obtained from the child health record books. In Poland, these two values are routinely measured during vaccination visits to the pediatrician and written down in the child’s health record book. Hence, it is possible to recreate the growth pattern in this early stage of a child’s development. According to the vaccination schedule, complete data of body mass from 2, 4, 6, 7, 13, and 17 months of life have been obtained. Measurements of body weight and height are also performed in Poland by pediatricians or nurses as part of the so-called balance sheets in children in 2, 4, 6, 8 and 10 years of age. Therefore, for each girl we have complete data on body mass performed as a part of vaccination procedures (2, 4, 6, 7, 13, 17 months), as well as data on body weight and height from balance sheets (2, 4, 6, 8 years) and from anthropometric measurements taken for the needs of this study (10, 11, 12, 13, 14, 15, 16 years). The final calculations included girls who did not miss more than two measurements of body height and weight.

### 2.4. Age at Menarche

On each examination of their body mass and height at school, girls were asked whether they had menarche. Given that the measurements were taken at annual intervals, participants were able to indicate the age at menarche with high accuracy. The final analysis included girls who experienced menarche and who remembered its date.

### 2.5. Body Mass Index

In girls, the body mass index (BMI), expressed by the formula BMI = [body mass (kg)]/[height (m)]^2^, is assumed to be a good surrogate for the level of body fat [42]. BMI was converted to age-specific BMI z-scores based on the World Health Organization (WHO) growth charts [43]. For the analysis, we used z-scores from BMIs calculated from height and weight measurements made at 8 years of age—considering this age as the prepubertal period—and from BMIs calculated at the date of menarche. To calculate the BMI at menarche, the value of the nearest measurements of body weight and height were subtracted from the last measurements proceeding the first bleeding. The result was then divided by the number of days between these two dates, and the quotient was multiplied by the number of days that had passed since the last measurements until menarche [14].

### 2.6. Catch-Up Growth

Based on the measurements from the child health record books, we determined the presence or absence of catch-up growth up to an age of two years. To evaluate infant growth, we calculated weight z-scores using the WHO growth charts [43]. The growth patterns of SGA children were classified as (1) no catch-up growth, if their growth parameters remained below the −2 SDS up to the age of two years, or (2) catch-up growth, indicating the achievement of height or weight at or above the −2 SDS.

### 2.7. Growth Curves and the Assessment of the Specific Parameters of Adolescent Growth Spurt

To determine the course of growth and to indicate specific parameters of adolescent growth spurt, we used the mathematical model of JPA2. This model provides a good fit for human growth [44]. The application of the model allowed us to determine with the greatest probability the real course of growth and to indicate specific parameters of adolescent growth spurt, such as the age at onset of adolescent growth spurt (age at take-ff, ATO), and age at peak height velocity (APHV) [44,45,46,47]. The JPA2 model is expressed by the formula [44]:

(1) $$y(t)=A\left\{1-\frac{1}{1+{\left(\frac{t+E}{D1}\right)}^{C1}+{\left(\frac{t+E}{D2}\right)}^{C2}+{\left(\frac{t+E}{D3}\right)}^{C3}}\right\}+E$$

where:

*t*—postnatal age
*Y*—height reached at age *t*
*A*—adult height
*E*—estimated prenatal duration of growth
*D*1, *D*2, *D*3—time-scale factors
*C*1, *C*2, *C*3—dimensionless exponents

### 2.8. Statistical Analysis

Calculations were performed in the Statistica 13 (STATSoft) program. The adolescent growth spurt parameters and growth curves were determined using the JPA2 model available in the AUXAL SSI 3.1 software. In order to calculate the differences between BMI, age at menarche, and parameters of adolescent growth spurt between the categories of girls distinguished due to their birth size and occurrence of catch-up growth, the Kruskal–Wallis test was used. Both the retrospective and status quo methods were used to determine the age at menarche in selected groups of girls. A *p*-value < 0.05 was considered statistically significant.

## 3. Results

From the total of 297 girls in the study, 77.4% were born AGA and 22.6% were born SGA. Catch-up growth had occurred in 65.7% of girls born SGA. The mean age at menarche for the entire group was 12.59 years (Me = 12.52, Min = 9.9, Max = 16.0), the mean age at start of adolescent growth spurt (TO) was 8.45 years (SD = 0.77), and the mean age at peak height velocity (PHV) was 11.47 years (SD = 0.74). At the time of TO, the girls’ mean body height was 131.26 cm (SD = 7.94), while the mean body height at PHV was 150.49 cm (SD = 6.61).

**Figure 2:**
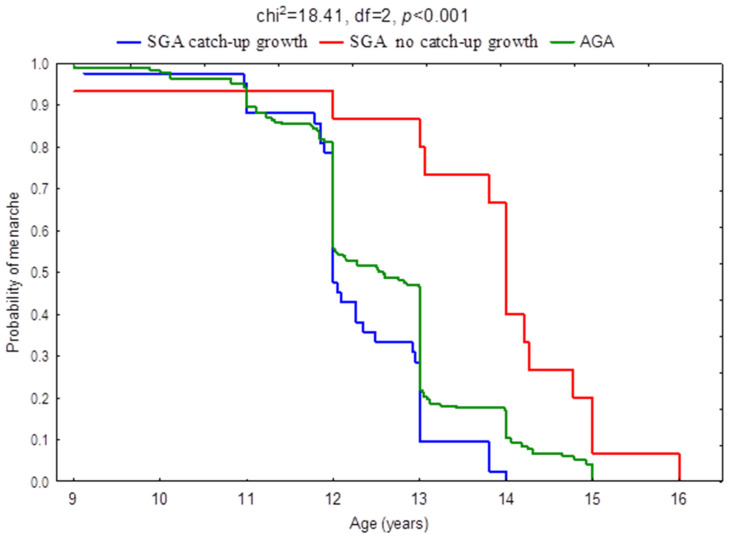
Kaplan–Meier curves applied to the age at menarche in girls born small for gestational age (SGA) with catch-up growth, in girls born small for gestational age (SGA) without catch-up growth, and in girls born appropriate for gestational age (AGA).

Statistically significant differences were found in all the analyzed variables between girls born SGA with and without catch-up growth and the control group (AGA). As presented in Table 2, girls born SGA in whom catch-up growth was observed showed the highest values of BMI at the age of 8 years (H = 94.22, *p* < 0.001) and at the time of menarche (H = 58.21, *p* < 0.001). SGA girls in which catch-up growth was not observed were characterized by the lowest BMI values in relation to the other analyzed groups. The age at menarche also differed significantly between the three designated groups of girls (H = 21.77, *p* < 0.001). The first menstrual bleeding occurred the earliest in girls born SGA with catch-up growth, and the latest in SGA girls without catch-up growth. Figure 2 presents Kaplan–Meier curves applied to the age at menarche in three selected groups of girls.

**Figure 3:**
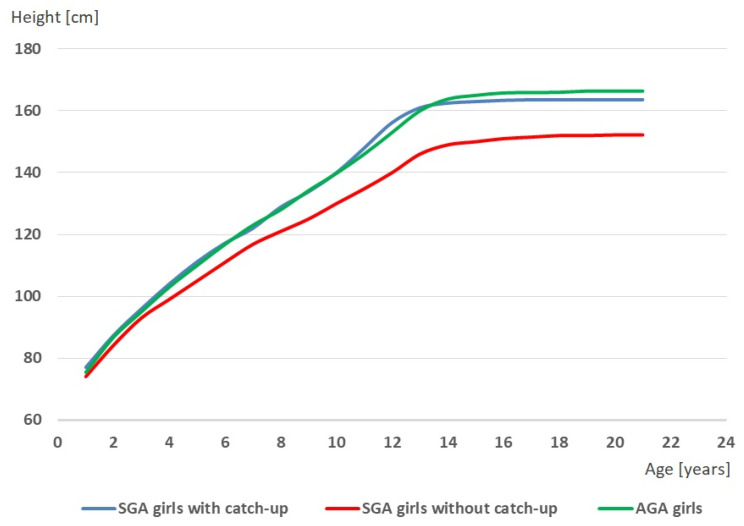
Growth curves of girls born small for gestational age (SGA) with catch-up growth, girls born small for gestational age (SGA) without catch-up growth, and girls born appropriate for gestational age (AGA).

**Figure 4:**
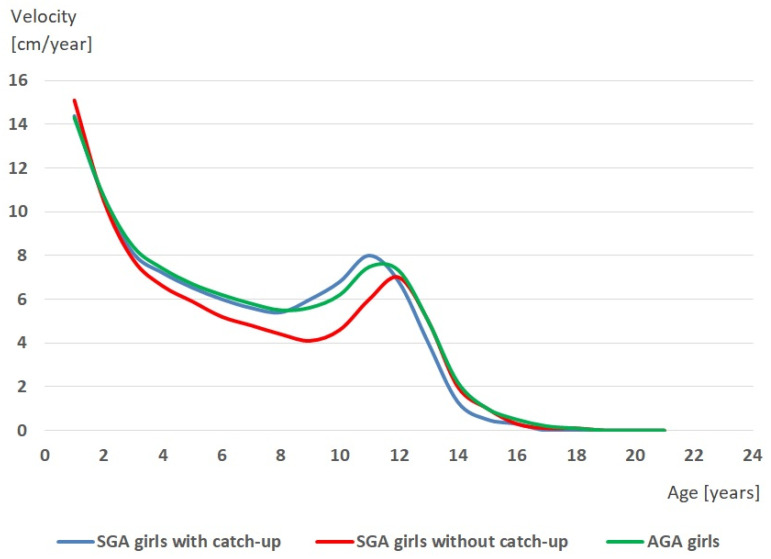
Growth velocity curves of body height of girls born small for gestational age (SGA) with catch-up growth, girls born small for gestational age (SGA) without catch-up growth, and girls born appropriate for gestational age (AGA).

It was also shown that girls classified into each of the three groups at a different age experienced an adolescent growth spurt of body height. The onset of the growth spurt (TO) occurred first in SGA girls with catch-up growth and the latest in SGA girls without catch-up growth (H = 6.54, *p* = 0.012). The peak height velocity (PHV) occurred the earliest in SGA girls with catch-up growth and the latest in SGA girls without catch-up (H = 11.71, *p* = 0.003). Thus, in girls born SGA in whom catch-up growth was observed, both the beginning and the peak of adolescent growth spurt occurred at the earliest in relation to AGA girls and SGA girls without catch-up growth. The latter girls, i.e., SGA girls without catch-up growth, experienced the growth spurt in relation to the other two groups of girls at the latest. Figure 3 and Figure 4 present the growth curves of body height (Figure 3) and growth velocity curves (Figure 4) of girls born SGA with catch-up growth, girls born SGA without catch-up growth, and girls born AGA.

## 4. Discussion

The results of this study clearly indicate that the occurrence of catch-up growth is a predisposing factor for a higher BMI in the pre-pubertal and pubertal periods as well as being associated with earlier puberty, measured both by age at menarche and age at growth spurt of body height. A comprehensive analysis of the long-term effects of catch-up growth or its absence on the physical development of SGA girls during puberty is still a rarity in the available literature and is the greatest strength of this study. The results of previous studies showed that SGA children are prone to have precocious pubarche (pubic hair development) and precocious adrenarche (increased secretion of androgens by the adrenal cortex), as well as an earlier entrance of puberty compared to their AGA peers [27,35,37,38,48,49]. It has already been shown by Hvidt et al. [30] that girls born with a low body weight according to their gestational age and who had 1-unit increase in z-score for body height within their first year of life had an early pubertal age compared to infants without catch-up growth. Moreover, SGA girls had menarche 1.6 to 2.3 months earlier when compared to girls born AGA. Similarly, in the study of Yadav and Rustogi [50], catch-up growth in SGA girls was associated with earlier menarche and faster puberty. In ALSPAC studies enrolling a large British cohort, children who showed rapid weight gain after birth had the highest levels of adrenal androgens and DHEA-S [51,52]. The attempts to explain this phenomenon boil down to the hypothesis proposed by Barker [10], according to which the low birth weight resulting from intrauterine malnutrition is a risk factor for the development of excessive fatness and insulin resistance in later life. The unfavorable conditions in the intrauterine environment may lead to physiological changes in the tissues of the developing fetus and to hormonal and lipid imbalance. The epigenetic patterns formed under the influence of physiological changes become permanent and may cause a variety of growth and health disorders in the postnatal life [53]. This risk seems particularly high when there is catch-up growth during infancy [54,55].

The link between excessive fatness and earlier puberty in SGA girls is most likely played by leptin, a hormone produced by adipose tissue. Leptin is considered a key factor in the central initiation of puberty as it has a direct effect on the secretion of gonadotrophins [56]. The leptin receptors have been found in kisspeptin-producing cells (kisspeptin plays an important role in the initiation of puberty) [57], in pituitary gonadotropin-producing cells [56,58], in ovarian follicular cells and in Leydig cells [15]. Considering that leptin levels correlate with levels of body fat, excessive adiposity may lead to premature activation of the GnRH pulse generator [59], and thus affect the timing and pace of puberty. In addition, excessive weight gain before puberty may cause a decrease in sex-hormone-binding globulin levels (SHBG), which in turn increases the bioavailability of sex steroids including estradiol [18]. Moreover, in adipose tissue, the process of aromatization of androgens to estrogens occurs [60]. It has also been shown that the high expression of aromatase in adipose tissue is associated with estrogen production from adrenal androgen precursors [51]. Additionally, the status of hyperinsulinemia/insulin resistance associated with a high BMI might promote the onset and progression of puberty. Hyperinsulinemia can stimulate the secretion of luteinizing hormone (LH) and lead to an increase in androgen levels, which in turn may promote pubertal development acting peripherally and/or centrally on the hypothalamic–pituitary axis [59]. Increased androgen levels in pre-pubertal girls can stimulate GnRH secretion, leading to an earlier onset of sexual maturation [18,61]. Thus, the percentage of body fat in the total body mass may be related to the rate of puberty in girls [62].

The results of the study showed that girls born SGA with catch-up growth experience the pubertal growth spurt the earliest in relation to AGA girls and SGA girls without catch-up. The main differences in the linear growth during puberty in SGA children compared to AGA children are based on accelerated skeletal maturation which may lead to earlier obliteration of the growth plate and lower final body height, the occurrence of an adolescent growth spurt in the earlier stage of sexual maturation—which may result in shortening the period of growth—and a less intensive adolescent growth spurt [4]. In this case, this relationship may be associated with a tendency to excessive fatness, which is significantly more common in SGA girls with catch-up growth. The results of previous studies have showed that, for children in the age preceding puberty, being overweight or obese accelerates linear growth [14,15,63,64]. Children with obesity have more receptors for growth hormone (GH), higher levels of insulin-like growth factor-1 (IGF-1), and higher levels of leptin and insulin [16,65,66]. Leptin interacts with GH and appears to stimulate the pituitary gland to secrete GH by inhibiting the production of somatostatin by the hypothalamus and increasing the secretion of GH through a direct effect on cells in the anterior pituitary that release GH [67]. Leptin can affect the level of sex steroids that regulate the secretion of GH and respond to this hormone peripherally [68]. It may act as a skeletal growth factor, able to stimulate both proliferation and differentiation of chondrocytes in the growth plate [18,69]. However, there are limitations to the published data. In available articles, the catch-up growth has been very rarely assessed, which means that SGA children were almost always treated as a homogeneous group.

There is another important aspect regarding the early maturation of SGA girls with rapid postnatal growth that cannot be overlooked. Generally, an earlier rate of pubertal maturation in girls correlates with a number of detrimental psychological, medical, sexual, and social outcomes compared with on-time or later maturation [70,71]. Interindividual differences in the timing of puberty create a period of contrast in which girls of the same age differ greatly in very significant physical characteristics, such as breast size, fat distribution, body proportions, or body hair [72]. SGA girls with catch-up growth, who tend to mature earlier than their peers, may find pubertal adjustment especially challenging [71].

The results of this study showed that girls SGA without catch-up growth in infancy had lower BMI values in the pre-pubertal and pubertal periods and entered puberty later than the SGA girls with catch-up or AGA girls. This may indicate that, in some SGA children, low birth weight is genetically determined regardless of maternal or environmental factors [73,74]. In SGA children with persistent growth failure, abnormal functioning of the GH-IGF-1 axis is usually observed. The published data show that more than half of SGA children without catch-up growth have a reduced 24-h GH profile and/or a low-stimulated GH [75,76]. GH resistance is common in SGA children with persistent short stature, but IGF-1 concentrations range from below normal to above normal [77,78]. There is a certain regularity that GH deficiency is observed in SGA children with low IGF-I concentration, while in SGA children with high IGF-I concentration there is a defect in the IGF receptor gene (*IGF1R*) [79,80]. Although delayed puberty and persistently reduced body height are sometimes treated as a completely benign disease entity in SGA children not requiring growth hormone or sex steroid treatment, they may result in lower bone mineral density, negative psychological outcomes, and increased risk of cardiovascular disease [81].

This study has some limitations. Body height measurements from the early stages of girls’ development were made by different medical professionals and using different equipment, which is associated with a large measurement error. Despite the standardized techniques used to measure body height from 10 to 16 years of age, previous data may have influenced the final values of the parameters of pubertal growth spurt of body height. Moreover, in the study we analyzed the relationship between the size at birth and parameters describing both the rate of sexual maturation (age at menarche) and the age of adolescent growth spurt of body height. Due to the lack of data on the age of puberty in boys, such as testicle size, we did not include this sex in the analyses. Additionally, since the study was not a clinical trial but was conducted in schools, we could not take blood samples to analyze the level of sex hormones, GH, IGF-1, or proteins binding these hormones, which significantly limited the interpretation of the results. We also did not have information on breastfeeding, which may have a modifying role in the physical growth of children.

## 5. Conclusions

Catch-up growth in girls born SGA is associated with higher BMI values in pre-pubertal and pubertal periods as well as with an earlier appearance of menarche and adolescent growth spurt of body height. Despite the positive aspects of the catch-up growth phenomenon, which allows children to compensate for a low birth weight, it has far-reaching consequences in the form of an increased risk of rapid fat accumulation and fast transition to puberty. For these reasons, the development of SGA girls, especially in the first years of life, should be carefully monitored.

## Figures and Tables

**Table 1:** The socioeconomic status of studied girls.

	N	%
Mother’s education		
Primary/vocational	78	26.26
Secondary	134	45.12
University	85	28.62
Father’s education		
Primary/vocational	134	45.12
Secondary	102	34.34
University	61	20.54
Number of children in the family		
One	107	36.03
Two	104	35.02
Three or more	86	28.95
Economic status of the family		
Good	109	36.70
Middle (average)	156	52.53
Bad	32	10.77

**Table 2:** Mean values of BMI at 8 ya and at menarche, age at menarche, and age at TO and PVH in categories of birth size and catch-up growth of the studied girls.

Variable	SGA, Catch-Up Growth	SD	AGA	SD	SGA, No-Catch-Up Growth	SD	H	*p*
BMI at 8 ya (z-scores)	1.49	1.19	0.31	0.95	−1.95	0.41	94.22	<0.001
BMI at menarche (z-scores)	1.47	1.10	0.45	0.97	−0.98	1.07	58.21	<0.001
Age at menarche (years)	12.25	0.88	12.57	1.16	13.86	1.60	21.77	<0.001
Age at TO (years)	8.28	0.77	8.57	0.63	8.72	0.79	8.85	0.012
Age at PHV (years)	11.36	0.77	11.55	0.72	11.89	0.85	11.71	0.003

H—the value of the Kruskal–Wallis test by ranks; *p*—significance value; ATO—age at take-off; APHV—age at peak height velocity; BMI—body mass index; SGA—small for gestational age; and AGA—appropriate for gestational age.

## Data Availability

The datasets obtained and/or analyzed during the current study are available from the corresponding author on reasonable request.
